# Internet of Underground Things in Agriculture 4.0: Challenges, Applications and Perspectives

**DOI:** 10.3390/s23084058

**Published:** 2023-04-17

**Authors:** Christophe Cariou, Laure Moiroux-Arvis, François Pinet, Jean-Pierre Chanet

**Affiliations:** Université Clermont Auvergne, INRAE, UR TSCF, 9 av. Blaise Pascal CS 20085, F-63178 Aubière, France

**Keywords:** Internet of underground things, wireless underground sensor networks, agriculture

## Abstract

Internet of underground things (IoUTs) and wireless underground sensor networks (WUSNs) are new technologies particularly relevant in agriculture to measure and transmit environmental data, enabling us to optimize both crop growth and water resource management. The sensor nodes can be buried anywhere, including in the passage of vehicles, without interfering with aboveground farming activities. However, to obtain fully operational systems, several scientific and technological challenges remain to be addressed. The objective of this paper is to identify these challenges and provide an overview of the latest advances in IoUTs and WUSNs. The challenges related to the development of buried sensor nodes are first presented. The recent approaches proposed in the literature to autonomously and optimally collect the data of several buried sensor nodes, ranging from the use of ground relays, mobile robots and unmanned aerial vehicles, are next described. Finally, potential agricultural applications and future research directions are identified and discussed.

## 1. Introduction

The agricultural sector is today facing to major economic, societal and ecological challenges [1,2]. In particular, the constantly rising food demand together with the diminution of arable lands due to a world population and an urbanization continually increasing puts today an important pressure on the agricultural production. This pressure is accentuated by a set of environmental concerns, as soil depletion, soil erosion, surface water pollution, ozone layer degradation and biodiversity loss. The problem is that the intensive farming practices, which enable us to reach high crop yields, contribute to these effects due to the massive use of phytosanitary products and heavy machinery. In addition, the global warming and climate change are becoming major concerns, especially for the water resource management and crop growth.

Agricultural practices and models have thus to be rethought to become more efficient, more productive and more environmentally friendly. Numerous actors and stakeholders agree today that a way to achieve this objective will come from the adoption of new emerging technologies such as the Internet of things (IoT), robotics, big data analytics, artificial intelligence (AI), cloud computing (CC), and blockchains, see [3,4]. As in the past, with the introduction of mechanization and automation in agriculture, the gradual introduction of these new technologies is leading to a new agricultural revolution, the fourth one, called Agriculture 4.0, see [5].

Data are at the heart of this digital revolution. Smart IoT devices are progressively implanted in the fields to feed decision-making processes with accurate environmental measurements (e.g., soil moisture, soil and air temperature, solar radiation, pH level, wind strength and direction), see [6,7,8,9]. In its simplest form, an IoT device is built around a microcontroller, a radio transceiver located aboveground and with more or less high communication range, a power source eventually recharged by a solar panel and one or several sensors connected. In most cases, the IoT devices are installed on the edge of the fields to not disturb the farming activities. The measurements, raw or preprocessed, are sent over the Internet and stored in databases by means of intermediate ground relays (e.g., gateways, cellular stations), satellite relays (e.g., nanosatellites at low Earth orbit (LEO) [10]) or collection vehicles (e.g., mobile robots, unmanned aerial vehicles (UAVs), [11]). Processing algorithms aggregate all the data collected with other information sources (e.g., historic of weather conditions, topography of the fields, soil type, satellite images) to determine the best actions to be performed at a specific location and a specific time (e.g., irrigation control, fertilizer application, seeding), see [9].

**Figure 1:**
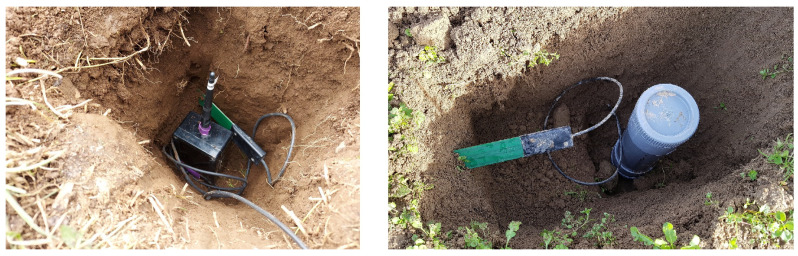
Examples of sensor nodes buried at 15 cm from the surface in a pasture with a moisture probe on the side [12]. The nodes will next be covered with soil and compacted.

However, in recent years and in complement to this approach, a new paradigm of IoT has emerged. This one is based on sensor nodes fully buried underground, including the radio transceivers and the antennas, at depths varying from a few centimeters to several tens of centimeters; see the examples in Figure 1.

That approach has led to the new concepts of Internet of underground things (IoUTs) and wireless underground sensor networks (WUSNs) [13,14]. The fact of burying and dissimulating the nodes underground has numerous advantages, such as the protection against the damage usually encountered by the aboveground nodes (e.g., theft, vandalism, degradation due to extreme climatic events). Moreover, the buried sensor nodes can be positioned anywhere, including on the passage of farm vehicles within the fields. However, before obtaining fully operational systems with long operational times (i.e., ideally several years without battery replacement), several scientific and technological challenges remain to be overcome, whether it be in terms of communication ranges, energy consumption or data collection. The objective of this paper is to identify these challenges and provide an overview of the recent advances of IoUTs and WUSNs for agricultural applications. To that end, this paper is organized as follows.

The research methodology used to identify and analyze relevant research articles on WUSNs and IoUT is first presented in Section 2. The current issues related to the development of buried sensor nodes are highlighted in Section 3. The approaches proposed in the literature to collect the data transmitted by the buried sensor nodes, whether it be from the use of intermediate ground relays, mobile ground robots or unmanned aerial vehicles (UAVs), are presented in Section 4. Some applications of WUSNs in Agriculture 4.0 and future research directions are identified and discussed in Section 5 and Section 6. The paper ends with the conclusions in Section 7.

## 2. Research Methodology

This paper provides a synthesis of the state of the art on wireless underground sensor networks (WUSNs) and Internet of underground things (IoUT) applied to smart farming. Guided by [15,16], we first formulated two research questions, namely, which approaches have been developed in the literature to build WUSNs and how the data can be collected from either ground relays, mobile robots or UAVs.

**Figure 2:**
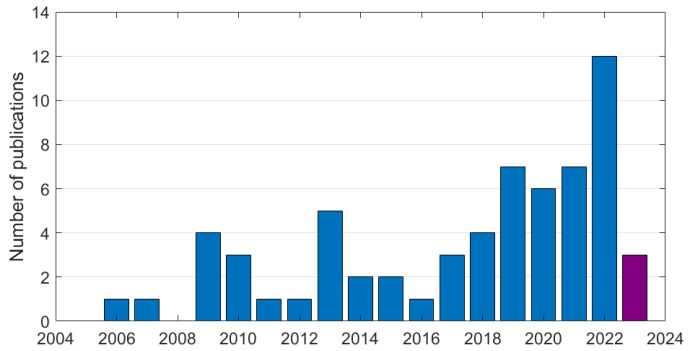
Distribution of the number of publications on WUSNs and IoUT by year.

We consulted the research databases “Scopus”, “Web of Science” and “IEEE Xplore” to identify the research articles. We limited the period of publication between 2000 and 2023, and limited our research to peer-reviewed journal articles and conference papers written in English. We searched the keywords “WUSN” or “Internet of Underground Things” in the titles; 75 papers were found by using this research strategy. After reading the abstracts, 12 papers were excluded from the review. We excluded, in particular, the studies applied to the monitoring of underground mines and tunnels as the sensor nodes are not directly in contact with the soil. Finally, 63 research articles, published from 2006 to 2023, were analyzed. Figure 2 presents the distribution of the publications by year. We can observe that, after the first publication in 2006, the number of publications increased regularly with an important growth from the year 2019 and a peak in 2022. The number of publications in 2023, which is the year of this analysis, continues to rise.

The relatively limited number of research articles found in the literature (75) suggests that the research on WUSN and IoUT is still in its early stage. To contrast with this result, 12759 research articles were found with the keyword “WSN”. The countries where the works are produced are presented on Table 1. We can observe that the United States comes first with the highest number of publications (23), followed by China (9), India (4), Saudi Arabia (4) and France (4). Table 2 classifies these publications by theme and subject (more details are given in Table A1, Table A2 and Table A3 in the Appendix A). Numerous works have focused on the development of propagation models of the electromagnetic waves in soil and also the study of routing protocols and the collection of data from static relay nodes.

Thirty-seven other research articles are cited in this paper relating to complementary subjects (e.g., Agriculture 4.0, smart farming based on IoT and UAV, agricultural robotics). In total, 100 works have been consulted and analyzed.

## 3. Challenges Related to the Development of Buried Sensor Nodes

**Figure 3:**
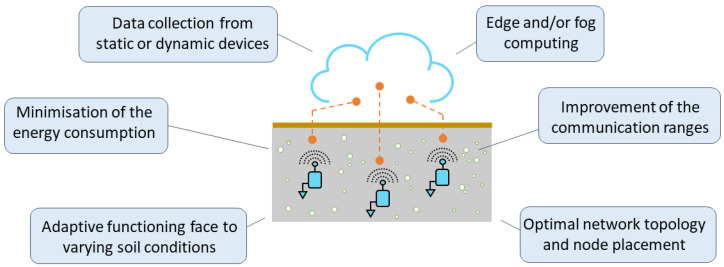
Main challenges related to the development of buried sensor nodes in a WUSN.

The development of wireless underground sensor networks (WUSNs) may appear to be very similar to their equivalent aboveground systems, the Wireless Sensor Networks (WSNs). However, the communication medium differs significantly, which requires deeply rethinking the existing architectures. In fact, the propagation of the electromagnetic waves is much more attenuated in the soil than in the air, about 20 to 300 times worse, see [17], which is a severe constraint for the underground communications. Moreover, the variations of the parameters of the soil medium, especially the volumetric water content (VWC), and the limited energy capacity of the buried sensor nodes with no possibility of recharge underground, are additional major and specific constraints. As a result, the network architectures developed for WSNs are not directly applicable to WUSNs. The current challenges related to the development of buried sensor nodes are highlighted in Figure 3 and discussed in the following subsections.

### 3.1. Adaptive Functioning Face to Varying Soil Conditions

**Figure 4:**
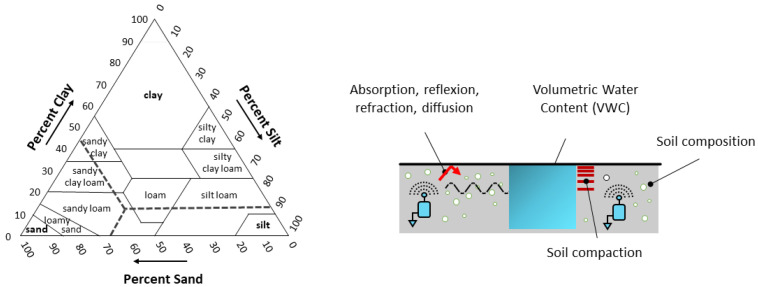
Soil classification represented by the United States Department of Agriculture (USDA), with the example of a sandy loam soil composed of 45% clay, 70% sand and 90% silt (**left**). Main factors impacting the propagation of electromagnetic waves in the soil medium (**right**).

As illustrated in Figure 1, the nodes of a WUSN are fully buried underground at a depth dependent on the application. When looking at a soil structure, it is composed of three main components, namely clay, silt and sand, each having different grain sizes (respectively, less than 0.002 mm, 0.002 to 0.05 mm, 0.05 to 2 mm); see the soil classification defined by the United States Department of Agriculture (USDA) in Figure 4 (left). The distribution of these three components determines not only the permeability of the soil to water and air but also its capacity of water retention (e.g., silt and clay soils have higher water-holding capacities than sandy soil). This water retention plays an important role in the communication ranges of the nodes as, in addition to being highly attenuated underground, the propagation of the electromagnetic waves is highly impacted by the soil moisture [21,22,23,24,61]; the more the volumetric water content (VWC) of a soil is high (i.e., the ratio of the volume of water to the volume of soil), the more the propagation of the electromagnetic waves is attenuated; see [25,26]. The issue is that the VWC of a soil varies spatially and over time, with respect to numerous factors (e.g., soil composition, ground topology, weather conditions, vegetation, human activities). That makes it difficult to estimate and predict the communication range of a buried sensor node at a specific time and a specific location. Moreover, as depicted in Figure 4 (right), many others factors impact the propagation of the signals in the soil medium. In particular, the absorption, reflection, refraction and diffusion phenomena lead to important signal losses. The intensity of these phenomena depends on the soil properties but also the burial depth of the sensor nodes. The compaction of the soil (bulk density) has also an important impact; the more a soil is compacted, the more the electromagnetic waves are attenuated, see [18,27,28,47].

Numerous works have focused on characterizing and modeling the propagation of the electromagnetic (EM) waves through the soil with respect to all these environmental factors impacting the signal propagation; see [29,30,31,32,33,37,51]. In particular, the Friis and Fresnel models have been proposed to model the signal path loss in soil. They are based on the knowledge of the complex dielectric permittivity of the soil. For example, considering a transmitter node and a receiver node, both buried underground, the Friis model enables us to calculate the received power $P_{r}$ with respect to the transmitted power $P_{t}$, the transmitter and receiver gains $G_{t}$ and $G_{r}$ and the total path loss underground $P_{lug2ug}$; see Equation (1). In this equation, the total path loss underground $P_{lug2ug}$ is defined with respect to the distance between the transmitter and the receiver $d_{ug}\left(m\right)$, a signal attenuation coefficient α and a phase shifting coefficient β; see Equation (2). The coefficients α and β are calculated from the frequency of the signal f(Hz), the magnetic permeability of the soil μ, and the real and imaginary parts of the dielectric permittivity (respectively $\epsilon^{\prime }$ and ϵ″); see Equation (3).

(1) $$P_{r}=P_{t}+G_{r}+G_{t}-P_{lug2ug}$$

(2) $$P_{lug2ug}=6.45+20\log\left(d_{ug}\right)+20\log\left(\beta \right)+8.69d\alpha$$

(3) $$\alpha =2\pi f\sqrt{\frac{\mu \epsilon^{\prime }}{2}\left(\sqrt{1+{\left(\frac{\epsilon \prime \prime }{\epsilon^{\prime }}\right)}^{2}}-1\right)}\;\;\;;\;\;\;\;\beta =2\pi f\sqrt{\frac{\mu \epsilon^{\prime }}{2}\left(\sqrt{1+{\left(\frac{\epsilon \prime \prime }{\epsilon^{\prime }}\right)}^{2}}+1\right)}$$

Several variants of this model have been proposed in the literature, in particular to take into account the burial depth of the sensor nodes and the reflection of the EM waves at the air-ground interface; see [38]. The underground-to-aboveground communications (UG2AG) and vice versa (AG2UG) necessitate also complementary models; see [39]. For example, the total pass loss of the UG2AG channel can be decomposed in three parts, namely the path loss underground $P_{lug2ug}$, the loss due to the refraction phenomena at the soil-to-air interface $P_{lug2ag}$ and the path loss aboveground $P_{lag2ag}$; see Equation (4). $P_{lug2ug}$ can be derived from Equation (1), $P_{refraction(ug2ag)}$ and $P_{lag2ag}$ from Equation (5); see [38] for more details.

(4) $$P_{lug2ag}=P_{lug2ug}+P_{refraction(ug2ag)}+P_{lag2ag}$$

(5) $$P_{refraction(ug2ag)}≃10log\frac{{\left(\sqrt{\epsilon^{\prime }}+1\right)}^{2}}{4\sqrt{\epsilon^{\prime }}}\;\;\;;\;\;\;\;P_{lag2ag}=-147.6+20\log\left(d_{ag}\right)+20\log\left(f\right)$$

Similarly, the total pass loss of the AG2UG channel can be decomposed in three parts, namely the path loss aboveground $P_{lag2ag}$, the loss due to the refraction phenomena at the air to soil interface $P_{lag2ug}$, and the path loss underground $P_{lug2ug}$; see Equation (6). $P_{lug2ug}$ and $P_{lag2ag}$ can be derived from respectively Equations (1) and (5), $P_{refraction(ag2ug)}$ from Equation (7); see [38] ($\theta_{I}$ is the incident angle of the EM signal).

(6) $$P_{lag2ug}=P_{lag2ag}+P_{refraction(ag2ug)}+P_{lug2ug}$$

(7) $$P_{refraction(ag2ug)}≃10\log\frac{{\left(\cos\theta_{I}+\sqrt{\epsilon^{\prime }-\sin^{2}\theta_{I}}\right)}^{2}}{4\cos\theta_{I}\sqrt{\epsilon^{\prime }-\sin^{2}\theta_{I}}}$$

An important work consists of comparing the results obtained with these models obtained in a theoretical or empirical manner with experimental measurements. Table 3 presents a few examples of these works. The issue is that the soil is a very complex and dynamic medium, and the estimation of signal attenuation is difficult to measure accurately due to numerous influencing factors. Moreover, to improve the accuracy of the models, the measurement of the complex dielectric permittivity of the soil at the location of the sensor nodes, e.g., based on time domain reflectometry (TDR) [33], is particularly important.

**Figure 5:**
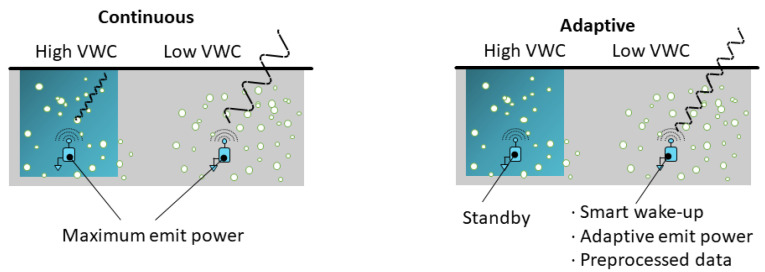
Two examples of operating modes: Continuous (**left**) and adaptive (**right**).

In addition, one of the main challenges in the development of reliable WUSNs is to take into account the limited and non-constant communication ranges of the buried sensor nodes, and adapt their functioning accordingly. Currently, the WUSNs proposed in the literature are mainly based on buried sensor nodes operating continuously, eventually combined with wake-up time windows and duty cycles; see Figure 5 (left). The emit power is usually tuned to the maximum value (+14 dBm/ 25 mW), and the buried sensor nodes are placed close to one other to maximize the connectivity to the detriment of the energy consumption and the number of nodes to be deployed. However, the future challenge will be to adapt the functioning of the buried sensor nodes to the soil conditions to minimize the energy consumption; see Figure 5 (right). In particular, when the VWC becomes too high, the node could limit its data transmissions to the minimum until to be set in standby mode. When the VWC decreases, the node could wake-up and transmit a limited number of preprocessed data with the minimal emit power to communicate with an aboveground node. Obviously, such operating modes should be determined together with the choice of the network topology and the placement of the nodes requested by the application [40,41].

### 3.2. Determination of the Network Topology and Node Placement

**Figure 6:**
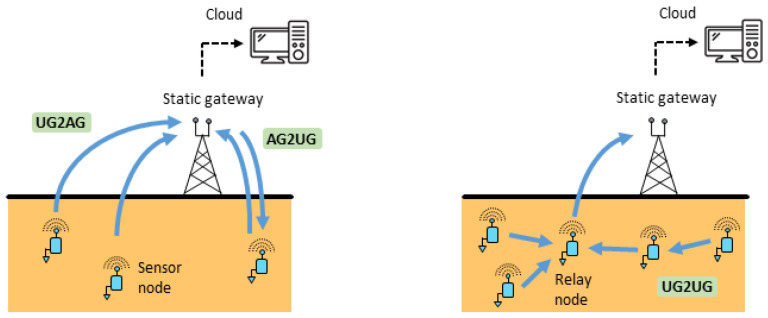
Two examples of topology in a WUSN: With independent buried sensor nodes (**left**) and with an intermediate relay node and UG2UG communications (**right**).

Three types of communication link can be present in WUSNs, namely underground to aboveground (UG2AG: the transmitting node is underground and the receiving node is aboveground), aboveground to underground (AG2UG: the transmitting node is aboveground and the receiving node is underground) and underground to underground (UG2UG: the transmitting and receiving nodes are both underground). The signals, however, do not propagate in the same manner according to the type of communication link [52]. On the one hand, the UG2UG communications between two buried sensor nodes are still difficult to obtain today for more than a few meters as the signal is completely propagated through the soil medium [34]. The WUSNs based on UG2UG links require, therefore, a high number of buried nodes to route the data, which can be relatively expensive and non-efficient. On the other hand, UG2AG and AG2UG communication links can reach longer distances (e.g., several hundreds of meters in certain conditions [12,62]), but they are not symmetrical: the UG2AG communications are usually of better quality than the AG2UG communications, see [72]. Based on these first findings, two main network topologies have been proposed in the literature [18,25], as depicted in Figure 6.

The simplest topology is based on independent buried sensor nodes; see Figure 6 (left). Each node communicates with an aboveground node (gateway) positioned at proximity by using UG2AG communications to transfer the data. AG2UG communications can also be implemented, for example, to update some parameters. This topology enables a rapid deployment of a star-shaped network with relatively reliable communications [25]. In counterpart, many gateways have to be installed aboveground, depending on the communication range of the buried sensor nodes and their distribution in the field. A second topology integrates one or several underground relay nodes; see Figure 6 (right). These nodes collect first the measurements of all the buried nodes in their network through UG2UG communications and multi-hop operations [42,48]. The data are next transmitted to the aboveground node (gateway) through UG2AG communications. This approach is more complex to develop as it requires the integration of routing protocols and different strategies (e.g., the role of a relay node can be given to another one to distribute the energy costs), [35,43,44]. This topology is also confronted by the difficulty to obtain reliable UG2UG communications and by the risk that the failure of one node can make all the network inoperative [45,77]. Future challenges on the network topology underground are thus mainly based on the improvement of the performances and reliability of the UG2UG communication links.

The development of a WUSN has also to consider the optimal number and location of nodes with respect to the agricultural needs and ground topology. That objective raises some problems of node distribution and data collection, all the more in large-scale farming and isolated environments [78]. The burial depth of the nodes can also be important in agriculture (e.g., more than 60 cm in the case of tillage operations). The minimization of the energy consumption of the buried sensor nodes is therefore essential to avoid costly and tedious maintenance operations.

### 3.3. Minimisation of the Energy Consumption of the Buried Sensor Nodes

**Figure 7:**
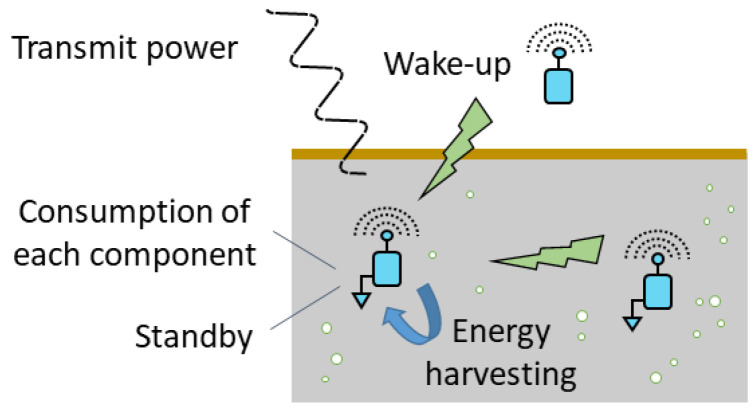
Different ways to minimize energy consumption of buried sensor nodes.

The buried sensor nodes can not be easily accessible, all the more when they are deeply buried below the surface. Moreover, contrarily to the WSNs, it is difficult to access energy recharge systems (e.g., solar panels). To make the deployment of a WUSN profitable, the minimization of the energy consumption of the buried sensor nodes is therefore a major challenge [46,49,79]. This one can be addressed in several ways, as highlighted in Figure 7.

First of all, the selection of each electronic device composing the node has to be determined carefully with respect to its energy consumption, and especially the radio transceiver which is the most energy consuming component, e.g., a node can consume only a few micro-amperes in deep-sleep mode and several tens of milli-amperes during a data transmission; see [80]. As seen previously in Section 2, the operating parameters can also be optimized to minimize both the activation phases (i.e., duty cycles) and the duration of the transmissions. These activation phases can be triggered by previously defining time windows but also by using a wake-up signal received from another node, located aboveground or underground. For example, Ref. [81] investigated the possibility to wake-up a sensor node located aboveground from an UAV by using either infrared or radio frequency signals. The development of wake-up systems for underground nodes is a real challenge which could open numerous perspectives for the development of WUSNs; see [50]. The development of energy harvesting solutions is another way to prolong the lifetime of the nodes. The principle consists of retrieving some energy from natural sources (e.g., sun, water, wind, heat [82,83,84,85]), radio-frequencies [86] or vibrations [87]. The aboveground nodes (gateways) can advantageously improve their autonomy with such an approach. The extension to the underground context is an interesting perspective to improve the efficiency of WUSNs in agriculture; see [47].

### 3.4. Improvement of the Communication Ranges

To obtain a reliable WUSN, it is essential to have nodes with high communication range capabilities, whether it be to communicate with each other or reach the aboveground nodes (gateways). The acoustic waves, magnetic induction (MI) and electromagnetic waves (EM) are the main technologies which have been investigated to communicate through the soil; see [19]. The acoustic waves can reach distances of a few tens of meters underground [36], but the very low data rate, high noise levels and delays limit their uses in WUSNs [30,75]. The technologies based on magnetic induction (MI) are limited in terms of communication distance of about a few meters [55,56,57,58,59]. Ref. [60] proposed to install some relays composed of small transmitter and receiver coils to ensure the magnetic waves continuity, but the accurate orientation of the coils is difficult to carry out in practice. The electromagnetic waves remain, therefore, the main communication technology used in WUSNs today. This technology enables us to communicate with higher data rates in comparison with acoustic and MI solutions and is easier to deploy in practice. However, the soil moisture and burial depth highly attenuate the propagation of EM. That leads to preferably limit the depth of the node to a few tens of centimeters. The UG2UG is also limited to a few meters. The low-power wide area networks (LPWAN) are generally used as they enable a low energy consumption of the buried sensor nodes with a good signal penetration; see [63,88]. For example, Ref. [12] succeeded in reaching a UG2AG communication link of a few hundred meters with sensor nodes buried between 15 to 30 cm deep using the technology LoRa [89].

The difficulty concerns, however, the selection of the operating frequency together with the antenna design. In fact, on the one hand, the phenomena of wave absorption by water is more important when the frequencies are high. It would be thus preferable to work with low-frequency ranges to improve the communication ranges. However, by decreasing the frequencies, the size of the antennas increases, which poses some problems of installation underground. On the other hand, the antennas designed to operate aboveground have their performance degraded underground. In addition, the operating frequency is more or less shifted with respect to the soil moisture [53]; the higher the operating frequency is, the more the shift downwards is important. For example, [54] observed that if the VWC increases from 5% to 40%, the operating frequency is shifted from 357 MHz to 146 MHz. The issue is that the soil moisture is not constant over time, varying with the weather conditions. Therefore, even if an antenna is specifically adapted for a type of soil and a given burial depth, the antenna will always have variations with respect to the VWC.

## 4. Data Collection of the Buried Sensor Nodes

**Figure 8:**
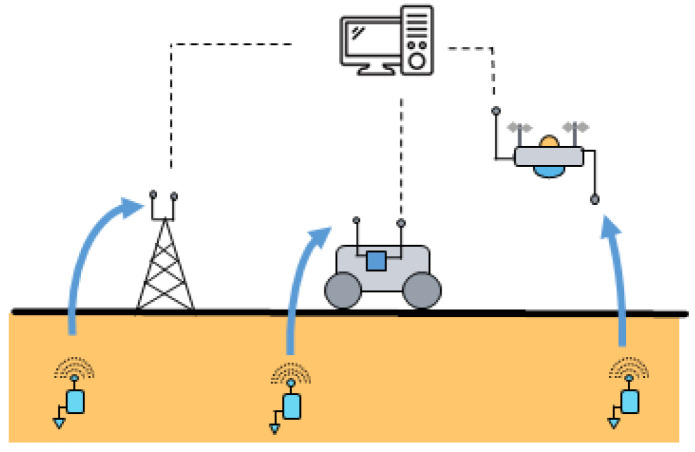
Static and dynamic ways to collect the data of buried sensor nodes.

After having presented the different challenges related to the development of buried sensor nodes, the following section will highlight the challenges related to the collection of the data transmitted. This collection task can be carried out by using static approaches (e.g., static ground relays) or dynamic ones (e.g., mobile robots and unmanned aerial vehicles (UAV)); see Figure 8.

### 4.1. Static Approaches

In the literature, the conventional approach to collect the data consists in installing ground static gateways in the fields, with advanced processing powers and communication capabilities, to relay the information transmitted by the sensor nodes towards the servers [76,87]. The problem is that the communication ranges of the buried sensor nodes can be limited: in [12], the maximal reachable horizontal distance of the communication link in LoRa at 868-MHz was 275 m by considering a sensor node buried at 15 cm deep and a gateway at a height of 2 m from the ground. Similarly, ref. [64] buried 23 sensor nodes at 40 cm depth within an area of 530 m radius. A static gateway, with an antenna mounted at a height of 10 m, was positioned in the center of the area. The communications were based on Sigfox at 901.2-MHz. The loss of data packets reached 50% when the horizontal distance between the node and the base station was 250 m and rapidly increased with respect to the distance. The drawback of this approach is therefore to require the multiplication of the number of gateways in the case of wide areas to be covered. It is, moreover, not cost-effective (e.g., cost of gateways, installation, maintenance operations), not flexible (e.g., difficult to rapidly change and/or extend an area of instrumentation) and energy consuming (the transmit power of the sensor nodes have to be high to reach the aboveground gateways). The aboveground infrastructure can, moreover, be subject to degradation due to weather conditions as well as malicious acts (vandalism, theft).

### 4.2. Dynamic Approaches

Another approach, still rarely investigated in the literature, consists of operating without the need of static gateways in the field. In this case, the data collection task is performed by using either a ground vehicle (e.g., mobile robot) or an unmanned aerial vehicle (UAV). These vehicles can embed a gateway connected to the Internet or, most of the time, simply a collector node storing temporarily the data collected.

**Figure 9:**
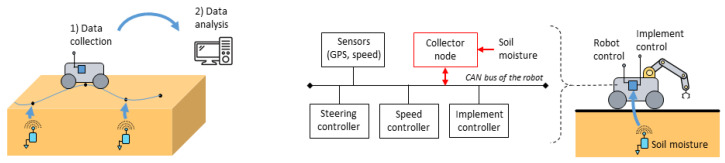
Data collection of buried sensor nodes by using a mobile robot (**left**). The soil moisture information is used in real-time to adapt the behavior of the robot accordingly [74] (**right**).

Agricultural robotics has developed strongly over the last two decades to increase farm productivity, perform environmentally friendly operations, as well as relieve farmers from tedious and unhealthy work [90]. When a connection to the cloud is available (e.g., from cellular networks, nanosatellites or long-range communication systems), robots are connected objects offering new possibilities in terms of data sharing and decision-making and also data collection; see [91]. In addition to their agricultural tasks carried out in the field (e.g., detection of weeds, pests and diseases; crop growth monitoring [92]), mobile robots could in the same time collect the data of the buried sensor nodes in the field; see the illustration in Figure 9 (left). This task could also be performed independently, requiring the definition of the trajectories to be followed beforehand. Ref. [72] was one of the first to consider such mobile nodes to collect the data of buried sensor nodes. In this work, different communication protocols were studied based on automatic re-transmissions and hand-shaking. Ref. [73] studied the use of a RFID reader embedded on a mobile robot to collect the soil moisture information estimated from the received signal strength indicator (RSSI) of passive RFID tags buried underground.

Recently, [74] proposed to not only collect and store the soil moisture information from buried sensor nodes but also use this information in real-time to adapt the behavior of the mobile robot (e.g., working speed, implement, trajectories); see Figure 9 (right). This approach is particularly interesting in the control of agricultural mobile robot as it enables facing varying weather conditions and/or heterogeneous soil status and optimizing both the locomotion of the robot as well as the agronomic task carried out. Moreover, that approach enables avoiding the situation where the robot is stuck and blocked in a wetland.

Another way to dynamically collect the data of buried sensor nodes is to use unmanned aerial vehicles (UAVs). This technology is increasingly used in smart farming to perform a great variety of missions, ranging from the monitoring of crop growth with multi-spectral cameras to the data collection of IoT devices [11,93,94,95]. In comparison to ground mobile robots, UAVs have clearly the advantage to be easily and rapidly operational on site. The flight time is, however, limited, which requires to optimize as much as possible the flight trajectories [96]. The regulations governing the use of UAVs in a country have also to be considered (e.g., in France, the use of an UAV in a professional context requires to pass theoretical and practical examinations beforehand. In case of flight out of sight, the maximal authorized horizontal distance is 1000 m).

**Figure 10:**
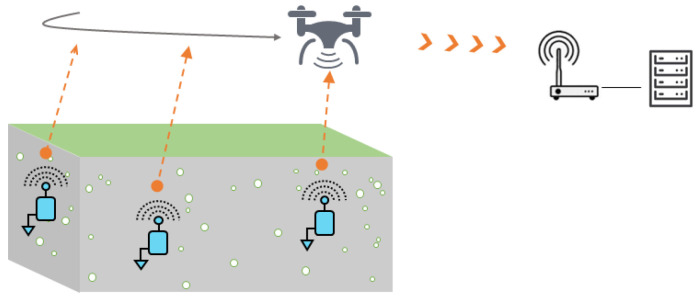
Data collection by means of a UAV: issues of optimal trajectory planning, reliable UG2AG data transmissions, data collection, data transfer on remote servers.

Still, very few works have investigated the data collection of buried sensor nodes from a UAV; see Figure 10. Recently, ref. [68] studied the impact of the altitude and lateral position of a drone on the received signal strength indicator (RSSI) by a sensor node buried at 15 cm deep in LoRa at 868 MHz. In addition to the experiments performed in hovering mode, dynamical flights enabled to collect data frames at the altitude of 40 m and with flying speeds of 4 m/s and 8 m/s. [69] characterized the decrease in the signal strength of LoRa communications with the increasing sensor node depth and flight height of the drone. Ref. [70] presented field tests carried out with a gateway mounted on an UAV, which received the soil moisture data transmitted by some sensor nodes buried at 30 cm deep. The communications were performed in LoRa at 916 MHz. The UAV flew at very low altitude, from 0.9 m to 3.6 m, with spiral trajectories. It was studied the packet loss at different horizontal distances and with different antenna orientations (co-polarized and cross-polarized). The results showed that less than 17% of the packets were lost at horizontal distances lower than 60 m whatever the relative orientation of the antennas. Ref. [71] presented simulations of a UAV flying in hovering mode above a potato crop of 20 hectares where 2000 sensor nodes were supposed to be buried. Based on NB-IoT communication technology, the objective of that work was to study the impact on the link quality of several parameters, such as the UAV altitude, the burial depth of the nodes as well as the soil moisture.

**Figure 11:**
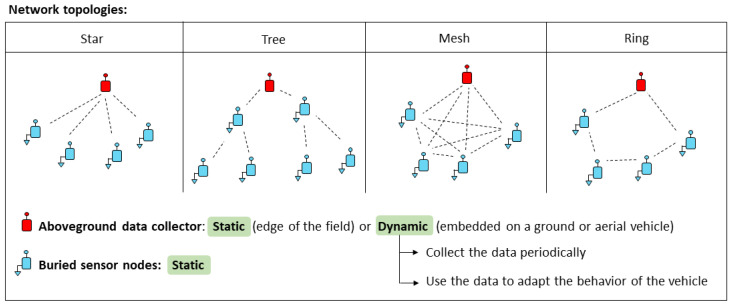
Overview of different possible configurations in terms of network topology and static or dynamic data collection.

To sum up the different possible configurations in the development of a WUSN, Figure 11 provides an overview of the overall system. Different network topologies can be considered underground (e.g., star, tree, mesh, ring or combining several types of these networks). The aboveground data collector can be static, usually installed at the edge of the field, but also dynamic. In this case, it is embedded on a ground vehicle (e.g., mobile robot) or aerial one (e.g., UAV). The data are collected periodically and successively. This approach provides flexibility. Moreover, the data can be used in real-time to adapt the behavior of the collection vehicle. Obviously, hybrid approaches could also be considered with static aboveground data collectors, storing the data until the passage of the collection vehicles.

## 5. Applications of WUSNs in Agriculture

**Figure 12:**
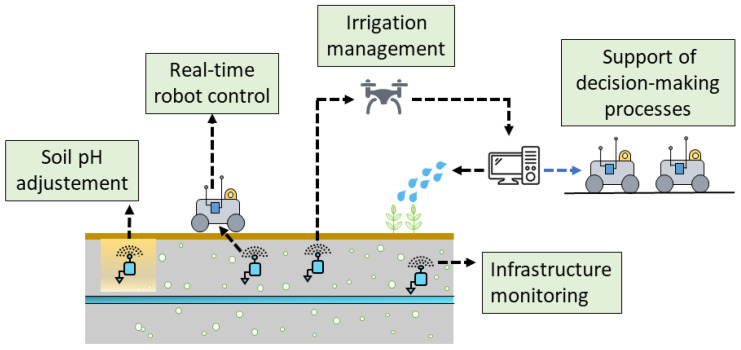
Fields of application of WUSNs in agriculture.

For the time being, the WUSNs have not yet been exploited on a large scale due to the panel of scientific and technical challenges listed in the previous sections and remaining to be tackled. However, WUSNs should progressively support numerous agricultural applications in the near future. In particular, the measurements of the soil moisture and temperature, accurately and at several depths, would enable the optimization of the irrigation processes and crop growth in the fields and also integrate decision-making processes as well as improve the behavior of autonomous vehicles in real-time; see Figure 12. Some part of these challenges are currently under development with the establishment of the first proof-of-concepts. The subsections hereafter present a brief list of these potential future applications.

### 5.1. Irrigation Management

The irrigation is the main agricultural application targeted for WUSNs in the literature. A well-managed irrigation system leads in fact to optimal crop yield and minimize the use of water. The introduction of buried sensor nodes, strategically placed in the field, would enable to regularly monitor the soil moisture and operate the irrigation process in closed-loop; see Figure 11. This objective is followed by several works; see [65,97]. In recent years, WUSNs have started to be implemented in real field conditions, mainly on corn crops. We can cite, for example, [64,66,67,69], who respectively used buried sensor nodes to optimize the irrigation in corn and soybean crops (continental climate; Illinois, USA), corn crops (continental climate; Nebraska, USA), poplar orchards (Mediterranean climate; California, USA) and corn crops (continental climate, Germany). The adoption and deployment of such smart irrigation systems will become essential in the near future to face the global warming and the increasing water scarcity.

### 5.2. Support of Decision-Making Processes

The germination, vegetative growth and yield performance of plants highly depend on the soil temperature and moisture. The knowledge of the dynamic of these parameters, in combination with other sources of information (e.g., weather conditions, ground topology, type of soil) would enable the precise determination of the optimal date for seeding and also the different times to perform the operations (e.g., tillage, fertilization; see [98]) and set the parameters of both the mobile robots and their implements (e.g., adjustment of the tyre pressure, depth of tillage, working speed). In addition, the tracking of the soil moisture and temperature data combined with the history of the field operations, the crop status and the plant varieties could enable us to accurately analyze the plant growth and develop optimal growth models. These data could also help to select the best varieties of plant and the optimal field operations accordingly, all the more in a climate change context.

### 5.3. Real-Time Robot Control

Currently, the accurate control of mobile robots is confronted to the incapacity to know the soil conditions beforehand, leading to issues of slippage, loss of traction and even blockage in wetlands. The soil conditions can, moreover, rapidly change following rainfalls or between the beginning and the end of the working day. The real-time measurements of the soil moisture at different depths could enable us to adapt in real-time both the locomotion (i.e., steering, speed) and the trajectories of the agricultural vehicles accordingly. Excessively wet areas could, for example, be avoided, with a re-planning of the trajectories to be followed by the robot. The settings of the implement could also be adapted in real-time to the soil moisture and temperature information measured in the field.

**Figure 13:**
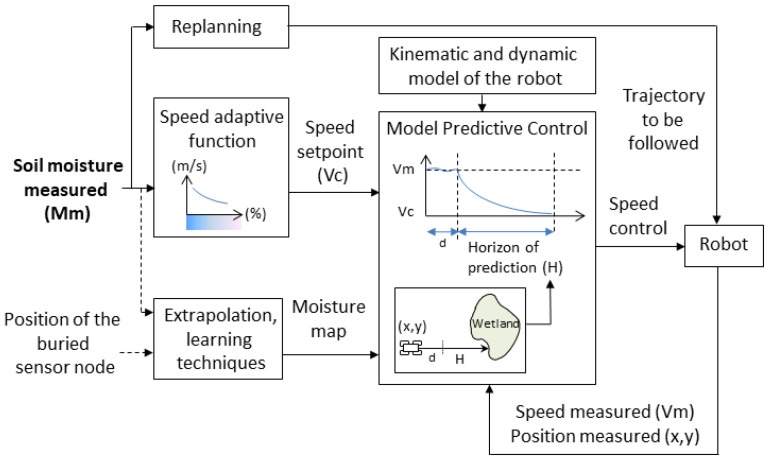
Use of the soil moisture information in the speed controller of a mobile robot [68].

Figure 13 presents an example of integration of the soil moisture information in the speed controller of a mobile robot. A speed-adaptive function determines the working speed of the robot with respect to the soil moisture measured. A model predictive controller (MPC) enables us to anticipate the speed variations while taking into account the kinematic and dynamic capabilities of the robot. The horizon of prediction is determined with respect to the current position of the robot and a moisture map progressively updated.

### 5.4. Infrastructure Monitoring

Another potential application of WUSNs is the monitoring of underground infrastructure in agriculture, such as pipes, drains and water storage tanks. In fact, pipes are widely used in agriculture to transport water pumped from a stream, well or reservoir to the point of use (e.g., crop, livestock watering), and the drains enable the removal of excess water from wet fields. WUSNs could be advantageously used to detect and locate pipe leakages and drain blockages, which is a particularly challenging and time-consuming task for the farmers. Leakages can, moreover, rapidly lead to substantial costs. It is therefore essential to monitor the good condition of the underground water network in farms and rapidly perform the repairs, all the more in the current context of water scarcity.

### 5.5. Soil pH Adjustment

WUSNs could also be used to monitor the fertility of the agricultural soils, in particular by measuring the soil pH. In fact, most of the crops requires a pH value ranging between 6 and 7 to grow properly. However, the pH of a soil changes over time and throughout the year with respect to various factors (e.g., agricultural practices, weather, type of crop). Limestone is commonly used by farmers to increase the pH of a soil, and sulfur is used to decrease the pH. Controlling the pH of the soil is therefore essential to enable optimal crop production, which can be brought by the development of buried sensor nodes and WUSNs.

## 6. Perspectives

The deployment of IoUT devices will certainly be significant in the near future, in particular in the agricultural fields to analyze, understand and support the consequences of the climate change on the crop growth and adapt the agricultural practices accordingly. However, that requires beforehand to overcome the different scientific and technical challenges highlighted in this paper, whether they be at the level of the buried sensor nodes or data collection processes.

One of the major remaining difficulties is to obtain reliable communications in WUSNs and develop buried sensor nodes having a long energy autonomy. The costs should also be limited (i.e., hardware, energy, maintenance, subscriptions for licensed communications if required). Moreover, the privacy and security issues should also be carefully studied [99,100]. In fact, the IoUT devices can not embed complex algorithms due to their limited memory and their constraint of energy consumption. They are thus particularly vulnerable to cyber attacks. Malicious people could, for example, take the control of the channels used for data transmission and capture, remove or modify the messages. The consequences can be important (e.g., modification of the data transmitted by moisture sensors leading to the flood of irrigated parcels). An important challenge of Agriculture 4.0 is therefore to secure with advanced algorithms each layer composing the IoUT (i.e., user, perception, data transport, data collection, data storage, data processing, actuation). That necessitates developing effective protocols enabling us to ensure, for example, data privacy (e.g., the data of sensors are confidential), location privacy (e.g., the positions of the sensors and mobile devices are not known), entity authentication (e.g., the transmitter and receiver must verify their identities before each data exchange), access control (e.g., the role and attributes of each entity is defined), authorization (e.g., each entity has a defined area of action) and data integrity verification. The pursuit of privacy and security goals is essential in new farming technologies and in IoUT in particular.

The optimal placement of the node is also a complex problem when the notions of priority, density of nodes, communication constraints and agronomic needs are considered together. Several WUSNs and WSNs can also co-exist, which raises some problems of interference. The optimal trajectory planning of the mobile data collector has also to be considered (e.g., solve the Close Enough Salesman Problem (CETSP), see [96]). Finally, all the data collected would require important storage capacities and the development of efficient management tools to analyze them and take the appropriate decisions.

## 7. Conclusions

This paper presented an overview of the main current scientific and technological challenges related to the development of WUSNs in agriculture. Several issues have been identified and discussed, ranging from the need of adaptive functioning of the buried sensor nodes face to varying soil conditions as ways to minimize energy consumption. Different approaches of data collection have been presented, based on either static ground relays, mobile robots or unmanned aerial vehicles (UAVs). A panel of potential applications was highlighted and several perspectives were pointed out.

WUSNs and IoUT will undoubtedly play a great role in the near future, especially in agriculture to contribute to the development of smarter and better targeted agricultural practices. The development of IoUT can lead to several changes as the creation of communities on the cloud regrouping farmers and experts in agronomy able to share experimental data on soil health and crop growth with other sources of information (e.g., weather prediction). The data collected can be used to train models (e.g., artifical intelligence) and deliver recommendations in real-time to farmers. Climate change will also certainly increase the demands of optimal management and automation of irrigation processes due to the increasingly scarcity of water. The development of IoUT can therefore lead to optimized crop production, improved productivity and reduced operational costs and also reduces food loss and providing the means to the autonomous vehicles to perform accurate and targeted work in the fields. However, an important challenge of IoUT will be to develop data exchange and communication standards to link the different entities together. That will contribute, moreover, to facilitate the adoption of these new technologies by farmers.

In addition, other sectors of activity would also benefit from the development of WUSNs, such as the monitoring of the environment (e.g., landslides, volcanic activities), the monitoring of infrastructure (e.g., stadiums [20]) and the detection of the passage of animals, vehicles and humans by using vibration sensors. All these applications require reliable communications, energy-efficient nodes and innovative data collection, which all represent exciting research directions. 

## Figures and Tables

**Table 1:** Classification of the number of publications on WUSNs and IoUT by country.

Continent	Countries (Number of Publications)
Africa	Ghana (2), Cameroon (1)
Asia	China (9), India (4), Saudi Arabia (4), Korea (1)
	Malaysia (1), Vietnam (1), Yemen (1)
Europe	France (4), Germany (3), United Kingdom (2)
	Finland (1), Italy (1), The Netherlands (1)
Oceania	Australia (2)
North America	United States (23)
South America	Brazil (1), Colombia (1)

**Table 2:** Classification of the publications by theme and subject.

Theme	Subject	References
WUSN	Concept	[13,17,18,19,20]
	Signal propagation	[21,22,23,24,25,26,27,28,29,30,31,32,33,34,35,36,37,38,39]
	Routing protocol	[40,41,42,43,44,45,46]
	Energy consumption	[47,48,49,50]
	Antenna	[51,52,53,54]
	Magnetic induction	[55,56,57,58,59,60]
Data	Static relay node	[12,61,62,63,64,65,66,67]
collection	UAV	[68,69,70,71]
	Mobile robot	[72,73,74]
IoUT	Survey, LoRaWAN	[14,75,76]

**Table 3:** Examples of model evaluation.

Ref.	Methodology	Models	Results
[22]	Laboratory, UG2UG	Empirical	- Validation of the model, $R^{2}\in \left[0.79;0.89\right]$
	433 MHz		
	Soil moisture 10–30%		
	Burial depth 30–70cm		
[23]	Onsite, UG2AG	Friis, Fresnel	- Better estimation with the Friis model
	915 MHz, 20–160 cm		
	2 soil compositions		
	Soil moisture 10–30%		
[27]	Laboratory, UG2AG	Semi-empirical	- Validation of the model, $R^{2}=0.91$
	2.44 GHz, 19 dBm,		- Need field evaluation
	2 soil compositions,		
	several moisture levels		
[29]	Laboratory, UG2UG	Friis, Fresnel	- Better estimation with Friis model in case
	434 MHz, 10 dBm,		of high moisture and high permittivity
	5 soil compositions,		- Better estimation with Fresnel model
	3 moisture levels		in case of low permittivity
[31]	Onsite, UG2AG	Theoretical	- Better results than with the Friis and
	433 MHz, 18.5 dBm		Fresnel models
	3 soil compositions		
	Soil moisture 0–48%		

## Abbreviations

The following abbreviations are used in this manuscript:

AG2UG	Aboveground to Underground
IoUT	Internet of Underground Things
LPWAN	Low-Power Wide Area Network
MI	Magnetic Induction
RFID	Radio Frequency Identification
RSSI	Received Signal Strength Indication
UG2AG	Underground to Aboveground
UG2UG	Underground to Underground
VWC	Volumetric Water Content
UAV	Unmanned Aerial Vehicle
WSN	Wireless Sensor Network
WUSN	Wireless Underground Sensor Network

## Appendix A

**Table A1:** Research works in IoUT.

Subject	Ref.	Methodology
Concept	[18]	- Present the research challenges in WUSNs
	[19]	- Present an overview of the architectures WUSNs
	[13]	- Highlight the future of WUSN
	[17]	- Present state-of-the-art, UG2UG, simulations and field experiments
	[20]	- Study the possibility to use WUSNs in stadiums
Propagation	[29]	- Evaluate Friis and Fresnel models for UG2UG at 434 MHz
	[30]	- Review the signal propagation models in soil medium
	[21]	- Propose a model on the Peplinski principle
	[27]	- Develop a semi-empirical model for UG2AG at 2.44 GHz
	[22]	- Design an empirical model for UG2UG at 433 MHz
	[31]	- Propose a theoretical model for UG2AG at 433 MHz
	[35]	- Review and compare propagation models
	[23]	- Evaluate Friis and Fresnel models for UG2AG at 915 MHz
	[34]	- Propose underground channel models
	[32]	- Evaluate a soil dielectric model
	[33]	- Compare the Friis and Fresnel models
	[28]	- Analyze the multi-carrier modulation underground
	[42]	- Develop an algorithm for detection of coverage hole
	[25]	- Evaluate the UG2UG communications
	[26]	- Develop a testbed for the communications underground
	[38]	- Analyze the inter-node connectivity in WUSN
	[14]	- Present underground channel models
	[36]	- Present a system based on acoustic waves
	[39]	- Develop and evaluate a path loss model
	[37]	- Study the propagation characteristic of EM waves
Routing	[43]	- Propose a routing protocol for WUSN
	[46]	- Optimize the power of the relay node on WUSN
	[44]	- Use cluster-based cooperative models
	[45]	- Deploy relay nodes to prolong network lifetime
	[40]	- Description of a single-hop approach in WUSN
	[41]	- Study the optimal placement of the relay nodes
Energy	[49]	- Analyze the energy consumption of different networks
	[50]	- Study the wake-up of buried sensor nodes
	[48]	- Propose multi-hoped communications guarantying energy efficiency
	[47]	- Present energy harvesting approaches for WUSN
Antenna	[53]	- Study the impacts of moisture on antenna return loss and bandwidth
	[54]	- Model the antenna return loss face to moisture variations
	[51]	- Present a design of phased array antennas
	[52]	- Study the impact of soil on UWB antenna
MI	[55]	- Analyze the multi-hop underground communications based on MI
	[56]	- Investigate MI communications based on relay circuits
	[57]	- Propose a routing protocol for WUSN based on MI
	[58]	- Compare the communications based on MI and EM waves
	[59]	- Present the challenges of MI communications in WUSN
	[60]	- Compare the communications based on MI and EM waves

**Table A2:** Research works in data collection.

Subject	Ref.	Methodology
Static relay	[64]	- Experiment 23 nodes in an agricultural field and predict soil VWC
	[65]	- Develop a UG2AG link to manage a pivot irrigation system
	[62]	- Evaluate LoRa at 433 MHz in UG2UG and UG2AG
	[66]	- Evaluate LoRa at 915 MHz in UG2AG
	[63]	- Evaluate UG2AG in LoRa in UG2AG
	[12]	- Evaluate UG2AG in LoRa at 433 and 868 MHz
	[67]	- Develop a UG2AG link to manage a pivot irrigation system
	[61]	- Experiment UG2AG in LoRa in different conditions
UAV	[68]	- Analyze the RSSI signals received by an UAV (high altitude)
	[71]	- Perform simulations with an UAV in hovering mode
	[69]	- Analyze the RSSI signals received by an UAV (low altitude)
	[70]	- Analyze the packet loss received by an UAV (low altitude)

**Table A3:** Research works in IoUT.

Subject	Ref.	Methodology
Robot	[73]	- Use RFID to collect soil moisture information
	[74]	- Control the speed of a robot from the soil moisture measurement
	[72]	- Develop communication protocols (re-transmissions, hand-shaking)
Survey	[76]	- Collect RSSI and VWC in LoRaWAN to develop a virtual VWC sensor
	[75]	- State-of-the-art on IoUT: challenges, technologies, applications
	[14]	- State-of-the-art on IoUT: challenges, technologies, precision farming
